# A Bio-Psycho-Social Approach to Understanding Optimism and Pessimism in Response to Stress

**DOI:** 10.3390/ejihpe14100176

**Published:** 2024-09-30

**Authors:** Yok-Fong Paat, Trina L. Hope, João B. Ferreira-Pinto, Hector Olvera Alvarez

**Affiliations:** 1 Department of Social Work, The University of Texas at El Paso, El Paso, TX 79968, USA; 2 Department of Sociology, University of Oklahoma, Norman, OK 73019, USA; 3 Dean’s Office, College of Health Sciences, The University of Texas at El Paso, El Paso, TX 79968, USA; 4 School of Nursing, Oregon Health & Science University, Portland, OR 97239, USA

**Keywords:** optimism, pessimism, coping, stress, outlook on life

## Abstract

Stress is widely known to have debilitating effects on physical health and mental wellbeing, particularly on one’s coping styles, personality traits, and outlook on life. Cumulative and chronic stress, which can serve as a triggering or aggravating factor for many pathological disorders if left unaddressed, has been linked to many life-threatening diseases. While many studies have looked at how optimism and pessimism are used as a form of coping mechanism, few have examined how different bio-psycho-social reactions to stress shape the level of optimism and pessimism. Using a sample of adult individuals aged 18 and older in the United States (n = 3361), this study addressed the following research questions: (1) What types of stress are predictive of optimism and pessimism? (2) Which responses to stress and coping mechanisms are most predictive of optimism and pessimism? (3) Do optimism and pessimism share the same stress-related risk and protective factors? Overall, this study found that while optimism and pessimism share conceptual similarities, they are not necessarily influenced by the same stress mechanisms. Stress, whether personal or financial, was associated with a negative outlook on life. This study showed that having good sleep quality and a lower number of psychological stress symptoms was linked to increasing optimism and reducing pessimism, while overeating or eating unhealthily was connected to both optimism and pessimism. Additionally, this study found that exercise/walking and emotional support mediated the effects of the responses to stress on the respondents’ level of optimism and pessimism.

## 1. Introduction

Optimism, conceptualized as the propensity to maintain a favorable outlook and positive orientation in the face of adversity, has received considerable attention in the literature, primarily as a predictor of more positive health and social outcomes [1,2]. Optimism has been linked to better coping skills, improved subjective wellbeing, a higher quality of life, and decreased mortality [3,4,5]. Pessimism, another type of distinct perceptual and cognitive trait characterized by negative viewpoints toward life, has been studied and compared/contrasted to optimism [6,7]. Specifically, pessimistic and optimistic individuals differ in how they cope with stress and how they are intrinsically motivated [8]. Unlike optimistic individuals, who are more likely to set goals, make plans, and anticipate positive outcomes, pessimistic people tend to experience more negative emotions (e.g., despair and sadness), focus on negative experiences/failures, and expect poorer outcomes [9]. These two modes of orientation are not necessarily independent traits; they have been regarded as a single construct, with each view characterizing the two ends of a single continuum [2,6].

Contemporaneously, stress is widely known to have a debilitating effect on physical health and mental wellbeing, particularly on one’s coping styles, personality traits, and outlook on life [10,11]. Cumulative and chronic stress, both of which can serve as a triggering or aggravating factor for many pathological disorders if left unaddressed, have been linked to many life-threatening diseases, including, but not limited to, heart disease, stroke, high blood pressure, diabetes, obesity, insomnia, body pain, cardiovascular diseases, hypertension, depression, and headaches [12,13,14]. While many studies have looked at how optimism is used as a form of coping mechanism in the recovery from cardiovascular disease, cancer, respiratory tract disease, spinal cord diseases, diabetes, and gastroenterological diseases (e.g., Crohn’s disease, ulcerative colitis) [15,16], few researchers have examined how different bio-psycho-social reactions to stress are linked to the level of optimism and pessimism. Using a sample of adults aged 18 and older in the United States, this study examined the stress-related bio-psycho-social factors linked to optimism and pessimism. We asked the following three research questions: (1) What types of stress are predictive of optimism and pessimism? (2) Which responses to stress and coping mechanisms are most predictive of optimism and pessimism? (3) Do optimism and pessimism share the same stress-related risk and protective factors?

## 2. Literature Review

### 2.1. Optimism and Pessimism

Optimism is linked to better self-rated health and positive experiences, including improved immunity, lower disease rates, and higher life expectancy [17,18,19]. There is evidence that optimistic people are more likely to take proactive steps to care for their health [9]. For instance, they are less likely to smoke, more likely to exercise and have healthy diets, and less likely to engage in behaviors that pose harm to their health [18,20], contrary to their pessimistic counterparts, who are more likely to engage in health-compromising behaviors [9]. Research also indicates that optimism is linked to reduced perceived stress and improved psychological functioning (e.g., better mental wellbeing) [21]. Specifically, evidence suggests that optimism can mitigate psychological distress, buffer the negative effects of stress, and moderate the adverse effects of life event stressors. In addition to reporting lower levels of distress and being less likely to experience negative emotions (e.g., hopelessness and depression) in response to stress, optimistic individuals are more likely to engage in goal-directed activity and less likely to experience psychopathology, onset/relapse, or mental illness [9].

While optimistic people engage in more constructive problem-solving and are better at managing conflicts, pessimists are more likely to engage in maladaptive behaviors [9,22]. Their reliance on different coping mechanisms can substantially impact life outcomes, particularly how they confront problems and deal with adversities. Optimistic people may be better at balancing expectations and pursuing their goals more effectively than pessimistic people, who are more likely to escape, avoid, give up, or stop trying [9]. There is evidence that optimists have more social connections and wider social networks than pessimists [9]. On the one hand, having social support is related to developing optimism [23] since people with positive expectations tend to be more readily accepted than those with negative ones [24]. On the other hand, optimists may experience more positive relationships and perceive greater social support [9]. Clearly, individuals who possess a more optimistic worldview benefit substantially across a variety of outcomes, as prior research suggests that optimism helps individuals deal with stress more effectively [9], but what contributes to the level of optimism and pessimism? Does the way in which individuals cope with stress affect their level of optimism and pessimism, given that stress, whether financial or personal, predicts a variety of poor outcomes [25,26]?

### 2.2. Responses to Stress and Coping Mechanisms

Stress is the body’s reaction to external threats. When faced with stress, the body responds by releasing hormones and activating various body systems that trigger a physiological response that prepares the body to fight or flee (also known as the “fight-or-flight” response). Stress is an integral part of our everyday life [27], but chronic stressors over a prolonged period increase the risks of long-term health complications by putting strain on crucial body systems, including nervous, respiratory, cardiovascular, gastrointestinal, and musculoskeletal systems [28]. Stress can also manifest in psychosomatic symptoms such as increased heartbeat, digestive problems, chronic fatigue, muscle tension, sexual difficulties, headache, sleep problems, and changes in appetite [28,29]. Sleep deprivation can exacerbate stress levels, while good sleep improves the capacity to handle stress more effectively [30]. Stress, whether repeated, acute, or chronic, can lead to the development of many mental health conditions [31]. Persistent stress increases the risks of long-term mental health challenges, including anxiety and mood disorders [31,32,33]. Emotional and psychological symptoms of daily stress also include increased agitation, anxiety, sadness, moodiness, anger, and low self-esteem [34]. Individuals who experience a stressful experience or are exposed to continuous or psychological trauma are more likely to develop pessimistic tendencies and be less resilient in the face of adversity [35]. Unavoidable stress can also contribute to a sense of learned helplessness, which may lead to feelings of anxiety and depression [36]. The transactional stress and coping model postulates that an individual’s capacity to cope is contingent upon their interaction with myriad personal and environmental factors [37]. Learning healthy ways to manage stress can be used as a coping mechanism to protect against stress, reduce negative feelings/symptoms, and increase the quality of life. Research suggests that physical activity can increase our brain’s production of endorphins, improving our sense of wellbeing [38]. Finally, while perceived stress can reduce life satisfaction [39], social support can serve as a vital resource to buffer stress [40].

### 2.3. Hypotheses

Based on the literature review, three distinct sets of hypotheses were proposed, each corresponding to one of our three research questions. First, this study hypothesized that respondents’ personal life stress (H_1a_) and economic stress (H_1b_) would be negatively related to their levels of optimism but positively associated with pessimism. Second, when examining the responses to stress, this study hypothesized that psychosomatic symptoms (H_2a_) and psychological stress (H_2b_) resulting from stress would be negatively related to optimism but positively related to pessimism. Further, having a good quality of sleep was expected to exert a protective effect against the respondents’ levels of pessimism and make optimism more likely (H_2c_), while eating too much or unhealthily due to stress was expected to increase pessimism but reduce optimism (H_2d_). With regard to coping mechanisms, this study predicted that respondents with emotional support would report higher levels of optimism but lower levels of pessimism (H_2e_). We also anticipated that respondents who utilized positive coping mechanisms, such as exercise or walking to manage stress, would be more optimistic and less pessimistic (H_2f_). Socio-demographics such as the respondents’ gender, age, race/ethnicity, nativity, marital status, educational attainment, and employment status were included in the study as control variables. These socio-demographic factors were incorporated to provide a comprehensive context for our study’s research questions. By controlling for these variables, our study aimed to isolate the unique contributions of stress and coping mechanisms in shaping individuals’ dispositions while accounting for potential variations across different demographic groups. This approach allowed for a more nuanced understanding of how stress-related factors influence optimism and pessimism across diverse segments of the adult population in the U.S. To address our third research question, we compared the two sets of variables that were statistically significant in our models predicting optimism (Model 3) and pessimism (Model 6). We hypothesized that the statistically significant variables in each model would differ (H_3_), allowing us to gain insights into distinct factors that influence each model’s outcomes.

## 3. Materials and Methods

### Data

The data utilized in this study came from “The Stress in America^TM^” survey, conducted online by the Harris Poll, a global market research firm, on behalf of the American Psychological Association. Data for this study come from the 2015 survey, collected in August 2015 in the U.S. Respondents were recruited from those who agreed to participate in the Harris Poll. Respondents were asked questions about their overall health and attitudes, stress, emotional support, the impact of stress, stress management, the outcome of stress they experienced, finances, and socio-demographics. A total of 3361 adult respondents took part in the survey in English, Spanish, Chinese, Vietnamese, and Korean [41]. The dataset can be accessible from the Inter-university Consortium for Political and Social Research (ICPSR) housed within the Institute for Social Research at the University of Michigan. We conducted secondary data analyses of this study, which qualifies as exempt from review by the University’s Institutional Review Board (IRB #: 1603003).

## 4. Measures

### 4.1. Dependent Variables: Optimism and Pessimism

In this study, optimism is operationalized as the respondents’ levels of hopefulness and anticipation for the best outcome for their future, while pessimism is referred to as the respondents’ expectations for the worst. Items used in this study came from the Life Orientation Test-Revised (LOT-R) [42] and have been found to be reliable and valid measures of optimism and pessimism [43,44]. Using a 5-point response scale ranging from “I disagree a lot” to “I agree a lot,” scales for optimism and pessimism were constructed by summing the scores of the selected items based on findings from the factor analyses and reliability analyses. Factor analysis is a statistical technique used to uncover patterns in the data and identify underlying dimensions that explain their patterns of correlations [45]. Cronbach’s alpha, used in reliability analysis, measures the internal consistency of the set of items for a scale, where higher values indicate that items are highly correlated with each other and measure the same underlying construct [46].

### 4.2. Stress and Responses to Stress

To identify significant sources of personal stress (e.g., health, relationships, discrimination) and economic stress (e.g., money, housing, job stability) in their lives and their degree of significance, respondents were asked to indicate their response using a 4-point response scale ranging from “not at all significant” to “very significant.” To measure psychosomatic reactions to stress, respondents were asked to report the symptoms they experienced in the past month due to stress (e.g., headaches, tightness in the chest, tooth grinding), along with a question about the quality of their sleep. In addition, the respondents were asked to identify psychological stress symptoms they experienced due to stress (e.g., forgetfulness, negative thoughts, constant worrying, and using a substance to relax). Finally, they were asked about unhealthy eating habits in response to stress.

### 4.3. Coping Mechanisms

To measure coping, respondents who indicated they had someone they could turn to for emotional support were coded 1; otherwise, they were coded 0. Additionally, those who exercised or walked to manage stress were coded 1, and those who did not were coded 0.

### 4.4. Socio-Demographic Variables

Males were coded as 1, and females as 0. Age was measured as a continuous variable. Black, Hispanic, and other racial groups are dummy variables for race/ethnicity, with White as the reference group in the multiple regression analyses. Other socio-demographic variables included nativity (foreign-born versus U.S.-born), marital status (married versus not married), college attainment (college graduate versus not college graduate), and employment status (employed full time versus not employed full time). Table 1 and Table 2 present detailed descriptions of the variables in the analyses and descriptive statistics.

## 5. Participant Characteristics

The participants had an average score of 10.9 for optimism and 8.2 for pessimism, ranging from 3 to 15. Additionally, the average score for personal life stress and economic stress were 11.8 (range: 5–20) and 16.6 (range: 6–24), respectively. On average, each participant experienced 3.9 psychosomatic symptoms and 3.7 psychological stress symptoms due to stress in the last month. About 52.3 percent of the participants rated their sleep quality as “good,” “very good,” or “excellent” in general. Approximately 42.7 percent of the participants reported that they overate or consumed unhealthy food due to stress in the last month. Further, about 70.4 percent of the participants felt comfortable asking for emotional support when needed, and 44.2 percent managed stress through exercise or walking. When socio-demographics were considered, about 33.0 percent of the participants were male, averaging 47.2 years old. With respect to race and ethnicity, about 32.0 percent were White, 24.7 percent were Black, 24.3 percent were Hispanic, and 19.0 percent belonged to other racial group. Close to two in ten participants (18.7 percent) were foreign-born. Close to half (49.3 percent) of the participants were married. Around 41.5 percent of the participants were college graduates, while about one-third (37.3 percent) were employed full time.

## 6. Analytical Approach

This study utilized multiple regression analyses to examine the empirical links between the respondents’ stress levels, responses to stress, coping mechanisms, and their levels of optimism and pessimism, with Full Information Maximum Likelihood as the estimation procedure. Multiple regression analyses are statistical procedures used to examine the relationship between a dependent variable and a set of independent variables in order to determine how the independent variables collectively explain the variations in the dependent variable [45]. Three models were constructed for each dependent variable. To establish the empirical link between stress with optimism and pessimism, the first models included the control variables and the two measures of stress (personal and economic). The second models added the responses to stress to determine if they mediated the effects of stress on optimism and pessimism. Finally, the third models added the two coping mechanisms to determine if emotional support and exercise/walking mediated the effects of stress or responses to stress on optimism and pessimism.

## 7. Results

Table 3 presents the results of the multiple regression analyses with optimism as the dependent variable. Respondents’ economic stress was negatively related to their levels of optimism in Model 1 (B = −0.073, *p* < 0.01). However, the variable lost its statistical significance in Model 2 with the inclusion of the responses to stress, suggesting that the responses to stress mediated the effects of economic stress on optimism. In Model 2, respondents who admitted to having a good quality of sleep were more optimistic than their counterparts who did not (B = 0.886, *p* < 0.001). Although the effect size was reduced slightly with the inclusion of the coping mechanism variables in Model 3, the statistical significance of sleep quality remained. The number of psychological stress symptoms was negatively associated with the respondents’ levels of optimism. In other words, for every additional symptom they reported, their score on the optimism scale decreased by 0.23 units (B = −0.234, *p* < 0.001 in Model 3). Model 3 introduced the two coping mechanisms. Contrary to expectations, unhealthy eating was positively related to the respondents’ levels of optimism in both Models 2 and 3 (B = 0.225, *p* < 0.05 in Model 3). Respondents who had someone for emotional support reported higher levels of optimism (B = 1.032, *p* < 0.001), as did respondents who exercised or walked to manage stress (B = 0.653, *p* < 0.001). The coping mechanism variables slightly mediated the effects of the statistically significant responses to stress variables, but all maintained their significance in Model 3.

With respect to the socio-demographics, age was positively associated with the respondents’ levels of optimism. For every one-year increase in age, the respondents’ levels of optimism increased by 0.020 units (B = 0.020, *p* < 0.001 in Model 3). Compared with their White counterparts, respondents who self-identified as Black or Hispanic and respondents who belong to other racial categories reported higher levels of optimism. Foreign-born respondents reported higher levels of optimism than their U.S.-born counterparts (B = 0.395, *p* < 0.01 in Model 3). Married respondents reported higher levels of optimism than non-married respondents (B = 0.281, *p* < 0.01 in Model 3). Both the college graduates and respondents in full-time employment reported higher levels of optimism (B = 0.297, *p* < 0.05 for college graduates and B = 0.274, *p* < 0.05 for respondents in full-time employment), but only in Model 1, suggesting that the effects of these two variables were mediated by the inclusion of the responses to stress variables.

Table 4 shows the results of the multiple regression analyses with the respondents’ levels of pessimism as the dependent variable. Both personal life stress and economic stress were statistically significant in Model 4 and remained so through to Model 6. When the three responses to stress variables were added in Model 5, the coefficient for personal life stress was slightly reduced, but the coefficient for economic stress was reduced by 50% (from 0.102 to 0.051), suggesting that half of the effects of economic stress on pessimism were explained by the individuals’ responses to stress. Among the responses to stress variables, respondents’ sleep quality was negatively related to pessimism in both Models 5 and 6 (B = 0.458, *p* < 0.001 in Model 6), while the number of psychological stress symptoms was positively related (B = 0.226, *p* < 0.001 in Model 6). Model 6 introduced the two coping mechanisms. Having emotional support was negatively associated with the respondents’ levels of pessimism (B = −0.916, *p* < 0.001). Similarly, exercise/walking exerted a protective effect, making pessimism less likely (B = −0.751, *p* < 0.001). While eating too much or unhealthily was not statistically significant in Model 5, it achieved significance in Model 6, suggesting that when accounting for coping mechanisms, respondents who ate too much or unhealthily in response to stress reported higher levels of pessimism (B = 0.263, *p* < 0.05 in Model 6). Like the prior analyses predicting the respondents’ levels of optimism, the responses to stress mediated the effects of economic stress, but the coping mechanisms did not mediate much of the effects of the responses to stress on pessimism.

When the socio-demographic variables were taken into consideration, males reported higher levels of pessimism compared with their female counterparts (B = 0.580, *p* < 0.001 in Model 6). In addition, with every one-year increase in age, this study found that the respondents reported lower levels of pessimism (B = −0.036, *p* < 0.001 in Model 6). In terms of race/ethnicity, Black respondents were less pessimistic compared with their White counterparts (B = −0.560, *p* < 0.001 in Model 6). Being in other racial groups (B = −0.362, *p* < 0.05) and married (B = −0.237, *p* < 0.05) were negatively related to their levels of pessimism but only in Model 4. Lastly, college graduates were less pessimistic compared with their non-college graduate counterparts (B = −0.374, *p* < 0.01 in Model 6).

## 8. Discussion

Stress is the body’s normal reaction to external events and manifests in various ways. On the negative side, it often results in a string of physiological, emotional, and psychological responses, where the body orchestrates a series of “fight-or-flight” reactions from perceiving threats to danger or survival [27]. This study aimed to examine the interconnections between stress, coping strategies, and the development of optimistic and pessimistic outlooks, focusing on identifying unique and shared factors influencing these dispositions. Using a bio-psycho-social approach to understand the roles that risk and protective factors for stress play in the level of optimism and pessimism traits, this study found that while optimism and pessimism share conceptual similarities, they are not necessarily influenced by the same stress mechanisms. This study found some evidence of stress reducing the respondents’ levels of optimism but increasing their levels of pessimism (H_1a_ and H_1b_). However, our study found that the participants’ psychosomatic symptoms had no statistically significant influence on optimism and pessimism (H_2a_). Further, having good sleep quality was linked to increased levels of optimism and reduced levels of pessimism (H_2c_), which aligns with research studies substantiating the findings that quality sleep is critical to maintaining optimal health. Specifically, epidemiological studies show that less sleep and poor sleep quality have been empirically linked to a broad spectrum of chronic and acute diseases, including depression, type 2 diabetes, and cardiac diseases [47]. Additionally, poor sleep can interfere with cognitive functions (including judgment and memory [30]), leading to loneliness and social isolation [48]. Uchino et al. (2017) stressed the importance of good health in the restorative process of good sleep [49]. Stress and sleep quality may reciprocally influence each other, as adults who sleep fewer than eight hours per night report higher levels of stress than their counterparts who had at least eight hours of sleep [30]. Kashani et al. (2010) found that participants with high stress levels experienced more daytime sleepiness and fatigue, had poorer sleep quality, and sleep less [50]. Therefore, it is not surprising that good sleep promotes optimism and reduces pessimism.

While more studies are needed to understand the type and severity of symptoms and respondents’ responses to stress, this study found that a greater number of psychological stress symptoms were connected to lower levels of optimism and higher levels of pessimism (H_2b_). Heightened mental health risks might be exacerbated by high stress levels and a negative outlook stemming from excessive worries about one’s health condition. Stressful experiences might also impede the respondents’ ability to handle life challenges. The evidence that optimism reduces the adverse effects associated with stressful life events related to mental disorder occurrence and re-occurrence is well established [51]. Mentally healthy individuals may be more likely to have a better outlook on life. Contrary to pessimism, which was empirically found to be linked to increased risks for depression and anxiety [52], optimism is negatively associated with depressive symptoms and suicidal ideation [53,54].

In contrast to one of our initial hypotheses that eating too much or unhealthily was expected to reduce optimism and increase pessimism (H_2d_), this study found that overeating or eating unhealthily increased both the respondents’ sense of optimism and pessimism. While the therapeutic effect of food on the respondents’ mechanism for coping could not be established in this study, emotional eating (i.e., eating in response to negative emotions), which is often associated with weight gain and obesity, is a common strategy in stress coping [55], where respondents might turn to food for comfort and/or suppress negative emotions. Ait-hadad and colleagues (2020), conversely, found that optimism is associated with the consumption of greater diet quality, including fruits and vegetables, and less snacking, indicating that examining the dietary intake and behavior over time may be fruitful in understanding adaptive coping styles and the development of dispositional optimistic attitudes [56].

There is evidence that under stressful circumstances, optimists reported stronger satisfaction in their relationships than their pessimistic counterparts [57]. This study found that respondents who reported greater access to emotional support were more likely to maintain a positive outlook and less likely to have a gloomy outlook than those who did not (H_2e_), underscoring the importance of relationship quality in the face of adversity. In this study, we found that exercising or walking to manage stress was linked to increased optimism and decreased pessimism (H_2f_), which aligns with the beneficial effects of exercise on mental health [58]. Specifically, exercise increases the body’s production of endorphins, the brain’s “feel-good” hormone [38], and improves self-esteem [59]. Empirical evidence has posited that exercise also reduces symptoms of anxiety, depression, and the adverse effects of stress [60]. Aside from the nonclinical populations, the benefits of exercise are extended to clinical populations. A meta-analysis by Giménez-Meseguer et al. (2020) found that exercise to manage stress alleviates symptoms of mental disorders and cravings and improves life quality among drug-dependent patients [61].

This study also found that men were more pessimistic than women. More work may be required to delve into the conceptual differences between optimism and pessimism, as the effect of optimism and pessimism may vary by population. Sha (2006), for instance, found that the negative effects of pessimism tend to increase for male college students who reported more stress [62], and our study corroborates that men appear to be more pessimistic than women. Meanwhile, Bjuggren et al. (2019) found that men are more optimistic than women about issues such as future economic situations [63]. When age was taken into account, this study found that the levels of optimism increased and pessimism decreased as the respondents aged. Lee et al. (2022) found that optimistic older adults might preserve their emotional wellbeing by engaging in emotion regulation strategies early on [64]. However, Chopik et al. (2015) showed that optimism typically increases before it decreases among older adults, an indication for future studies to investigate the curvilinear effect of age on the level of optimism and pessimism [17].

When race/ethnicity was considered, this study found that Black respondents were more optimistic than White respondents and less pessimistic than White respondents. Black individuals living in poverty, in particular, have been posited to be more optimistic than their White counterparts, indicating that optimism among Blacks does not stem from having a sense of security about their future [65] but rather their tendency to perceive their lives as better than earlier generations [66]. In this study, Hispanic and foreign-born respondents reported higher levels of optimism than their White and U.S.-born counterparts, respectively. Many Latinos hold a strong belief in the American dream and are optimistic about their future [67]. The optimistic immigrant theory postulates that recently arrived immigrants have high hopes for their future [68]. In addition, the healthy immigrant paradox noted that immigrants have relative health advantages, particularly recent immigrants, despite their lower socio-economic status [69]. Other socio-demographic variables, such as marital status and educational attainment, were also statistically significant predictors. Married couples’ access to their spouses’ support and the respondents’ need to secure a college degree in today’s competitive society, which is now perceived as a key to securing a job, presumably might have shaped their level of optimism. Overall, this study found that optimism and pessimism are not necessarily influenced by the same stress mechanisms or share the same stress-related risk and protective factors (H_3_).

## 9. Limitations

Some of this study’s limitations are worth noting. First, like any other study, this study is subject to social desirability bias, where the respondents might be more likely to provide survey responses that align with perceived socially acceptable norms. Second, this study only captured the respondents’ perceptions at one-time point due to the cross-sectionality of the data. The respondents’ disposition for optimism and pessimism might shape their perception of health and adaptive behaviors due to stress over time. Further, this study may be subject to self-selection bias, where optimistic people may be better at not engaging themselves in stressful situations than pessimistic individuals. Because the data were collected prior to the COVID-19 pandemic and given the evolving public reactions to stress [70], the findings of this study should be interpreted in light of this caution. Overall, this study shows that it is critical to understand the vulnerability to stress using the risk and resilience framework, particularly considering the importance of healthy coping mechanisms and the potential mediating effects of stress responses on optimism and pessimism. Thus, it is critical for public health and mental health practitioners to strengthen the promotion of optimism in their interventions and advocate the use of coping strategies that focus on both improving stressful situations and encouraging healthy lifestyles in their health promotion outreach and practice.

## 10. Implications

Stress can generate a wide range of physical and psychological responses, manifesting through personal and financial challenges, as indicated by our study. Our study showed that alleviating stress-related symptoms and enhancing stress-coping skills have the potential to increase optimism and reduce pessimism across adult life courses. These findings have significant implications for various professional and health settings. Healthcare professionals, including clinical psychologists, primary care physicians, and nurses, should consider incorporating optimism-building techniques into their practice [71,72]. This could involve cognitive restructuring exercises, stress level screenings, and brief interventions promoting stress management and optimistic thinking. Mental health centers may also consider increasing the presence of psychologists specializing in stress management and positive psychology, offering group therapy programs and workshops focused on building resilience and optimism [73,74].

In educational settings, programs to teach stress-coping skills and cultivate optimism from an early age may be a great option for implementation with school psychologists and university counseling centers. Workplace interventions, led by corporate psychologists and human resources departments, may consider designing and implementing stress management programs tailored to the specific challenges of the work environment [75]. Community centers and leisure facilities can play a crucial role by offering classes on stress management and positive thinking, while public health initiatives may develop awareness campaigns and easily accessible resources for stress reduction and optimism building.

Specific interventions based on our study results should focus on enhancing sleep quality, promoting physical activity, strengthening social support networks, addressing healthy eating habits, and managing psychological stress symptoms. For instance, given our finding that good sleep quality is linked to increased optimism, sleep hygiene techniques should be emphasized in varied clinical interventions. Similarly, this study’s revelation that exercise/walking mediates the effects of stress on optimism suggests that regular physical activity should be prescribed as part of stress management strategies. Finally, policies focusing on coping mechanisms such as increased social support and exercise promotion seem to hold promise based on our findings. By implementing these strategies across various sectors, we can create a multi-faceted approach to stress reduction and optimism promotion. This comprehensive strategy has the potential to not only improve individual wellbeing but also to enhance community mental health and reduce the societal burden of stress-related illnesses, creating a self-reinforcing cycle of reduced stress and improved optimism.

## 11. Conclusions

Optimism and pessimism have traditionally been viewed as opposite ends of a single continuum, yet they are not necessarily influenced by the same stress mechanisms. Stress, whether personal or financial, was associated with a negative outlook on life. This study showed that having good sleep quality and a lower number of psychological stress symptoms was linked to increasing optimism and reducing pessimism while overeating or eating unhealthily was connected to both respondents’ disposition toward optimism and pessimism. Additionally, this study also found that exercise/walking and emotional support mediated the effects of the responses to stress on the respondents’ level of optimism and pessimism.

## Figures and Tables

**Table 1:** Description of variables in analyses.

Variables	Sample Items	Response Format	Alpha
Optimism	Please indicate the extent to which you agree or disagree with the following statements:	5-point response scale from “I disagree a lot” to “I agree a lot”	0.83
	1. In uncertain times, I usually expect the best		
	2. I’m always optimistic about my future		
	3. Overall, I expect more good things to happen to me than bad		
Pessimism	Please indicate the extent to which you agree or disagree with the following statements:	5-point response scale from “I disagree a lot” to “I agree a lot”	0.83
	1. If something can go wrong for me, it will		
	2. I hardly ever expect things to go my way		
	3. I rarely count on good things happening to me		
Stress			
Personal life stress	For each one, please indicate how significant a source of stress it is in your life	4-point response scale from “Not at all significant” to “Very significant”	0.80
	1. Personal health concerns		
	2. Relationships (e.g., spouse, kids, girl/boyfriend)		
	3. Health problems affecting my family		
	4. Personal safety		
	5. Discrimination		
Economic stress	For each one, please indicate how significant a source of stress it is in your life:	4-point response scale from “Not at all significant” to “Very significant”	0.85
	1. Money		
	2. Work		
	3. Family responsibilities		
	4. Housing costs (e.g., mortgage or rent)		
	5. The economy		
	6. Job stability		
Responses to stress			
Number of psychosomatic symptoms	Which of the following, if any, have you experienced in the last month as a result of stress?	Counts of symptoms	
	Headache; Upset stomach or indigestion; Feeling as though I could cry; Muscular tension; Tightness in my chest; Feeling nervous or anxious; Feeling depressed or sad; Irritability or anger;		
	Lack of interest, motivation or energy; Fatigue; Teeth grinding; Feeling faint or dizzy; Change in sex drive; Change in appetite		
Sleep quality	In general, how would you describe the quality of your sleep?	1 = “Good,” “Very good,” and “Excellent”; 0 = “Poor” and “Fair”	
Number of psychological stress symptoms	Which of the following, if any, have you experienced in the last month as a result of stress?	Counts of symptoms	
	1. Forgetfulness		
	2. Inability to concentrate		
	3. Difficulty making decisions		
	4. Negative thoughts		
	5. Constant worrying		
	6. Agitation, inability to relax		
	7. Feeling overwhelmed		
	8. A sense of loneliness and isolation		
	9. Changes in sleeping habits (e.g., oversleeping, difficulty falling asleep, night waking)		
	10. Isolating yourself from others		
	11. Procrastinating or neglecting responsibilities		
	12. Using alcohol, cigarettes, or drugs to relax		
	13. Nervous habits (e.g., nail biting, pacing)		
	14. Harming myself (e.g., cutting, piercing, or hurting yourself)		
	15. Changes in my financial behaviors (e.g., overspending, dipping into savings, missing payments/bills)		
Ate too much or unhealthily	During the last month, did you ever eat too much or eat unhealthy foods because you were feeling stressed?	Yes = 1; No = 0	
Coping mechanisms			
Had someone for emotional support	Is there someone you can ask for emotional support if you need it, such as talking over problems or helping you make a difficult decision?	1 = Yes; 0 = No or “I don’t need help”	
Exercise or walk to manage stress	Do you do any of the following to manage stress? Exercise or walk	Yes = 1; No = 0	
Socio-demographics			
Male	How do you describe yourself?	1 = Male; 0 = Female	
Age	What is your age?	Age in years	
Race/ethnicity			
White	Respondent is White	1 = White; 0 = Non-White	
Black	Respondent is Black	1 = Black; 0 = Non-Black	
Hispanic	Respondent is Hispanic	1 = Hispanic; 0 = Non-Hispanic	
Other race	Respondent belongs to other racial category	1 = Other race; 0 = Non-other race	
Foreign-born	Respondent is foreign-born	1 = Foreign born; 0 = U.S.-born	
Married	Recoded from “What is your marital status?”	1 = Married or civil union; 0 = Other status	
College graduate	Recoded from “What is the highest level of education you have completed?	1 = College graduate; 0 = Not a college graduate	
Employed full time	Recoded from “Which of the following best describes your	1 = Employed or self-employed full time	
	employment status?”	0 = Not employed full time	

**Table 2:** Descriptive statistics.

Variables	Min	Max	Mean	SD
Optimism	3	15	10.902	2.941
Pessimism	3	15	8.244	3.384
Stress				
Personal life stress	5	20	11.759	3.850
Economic stress	6	24	16.621	4.624
Responses to stress				
Number of psychosomatic symptoms	0	14	3.913	3.598
Sleep quality	0	1	0.523	0.500
Number of psychological stress symptoms	0	15	3.677	3.744
Ate too much or unhealthily	0	1	0.427	0.495
Coping mechanisms				
Had someone for emotional support	0	1	0.704	0.457
Exercise or walk to manage stress	0	1	0.442	0.497
Socio-demographics				
Male	0	1	0.330	0.470
Age	18	92	47.173	17.826
Race/ethnicity				
White	0	1	0.320	0.467
Black	0	1	0.247	0.431
Hispanic	0	1	0.243	0.429
Other	0	1	0.190	0.392
Foreign-born	0	1	0.187	0.390
Married	0	1	0.493	0.500
College graduate	0	1	0.415	0.493
Employed full time	0	1	0.373	0.484

**Table 3:** Multiple regression analyses with optimism as the dependent variable.

	Model 1			Model 2			Model 3		
Variables	B		SE	B		SE	B		SE
Intercept	9.145	***	0.332	9.097	***	0.324	8.155	***	0.324
Stress									
Personal life stress	0.022		0.022	0.037		0.021	0.038		0.020
Economic stress	−0.073	**	0.022	−0.006		0.022	−0.007		0.021
Responses to stress									
Number of psychosomatic symptoms				0.012		0.026	−0.004		0.025
Sleep quality				0.886	***	0.104	0.749	***	0.102
Number of psychological stress symptoms				−0.245	***	0.024	−0.234	***	0.024
Ate too much or unhealthily				0.268	*	0.110	0.225	*	0.108
Coping mechanisms									
Had someone for emotional support							1.032	***	0.100
Exercise or walk to manage stress							0.653	***	0.091
Socio-demographics									
Male	−0.049		0.108	−0.162		0.102	−0.033		0.100
Age	0.032	***	0.003	0.019	***	0.003	0.020	***	0.003
Race/ethnicity (Reference = White)									
Black	1.245	***	0.137	1.079	***	0.129	1.088	***	0.126
Hispanic	0.850	***	0.150	0.790	***	0.141	0.840	***	0.137
Other	0.366	*	0.157	0.304	*	0.147	0.324	*	0.144
Foreign-born	0.636	***	0.144	0.360	**	0.136	0.395	**	0.133
Married	0.529	***	0.104	0.374	***	0.098	0.281	**	0.096
College graduate	0.297	*	0.107	0.156		0.100	0.080		0.098
Employed full time	0.274	*	0.108	0.171		0.101	0.154		0.099
R-squared	0.087			0.193			0.233		

* refers to *p* < 0.05, ** refers to *p* < 0.01, *** refers to *p* < 0.001. B = unstandardized coefficients; SE = standard error; n = 3361.

**Table 4:** Multiple regression analyses with pessimism as the dependent variable.

	Model 4			Model 5			Model 6		
Variables	B		SE	B		SE	B		SE
Intercept	7.263	***	0.357	7.035	***	0.353	7.933	***	0.355
Stress									
Personal life stress	0.166	***	0.024	0.140	***	0.022	0.139	***	0.022
Economic stress	0.102	***	0.024	0.051	*	0.023	0.051	*	0.023
Responses to stress									
Number of psychosomatic symptoms				−0.050		0.028	−0.034		0.028
Sleep quality				−0.620	***	0.114	−0.485	***	0.112
Number of psychological stress symptoms				0.237	***	0.026	0.226	***	0.026
Ate too much or unhealthily				0.227		0.121	0.263	*	0.119
Coping mechanisms									
Had someone for emotional support							−0.916	***	0.110
Exercise or walk to manage stress							−0.751	***	0.100
Socio-demographics									
Male	0.579	***	0.116	0.700	***	0.112	0.580	***	0.110
Age	−0.048	***	0.004	−0.035	***	0.003	−0.036	***	0.003
Race/ethnicity (Reference = White)									
Black	−0.707	***	0.147	−0.548	***	0.141	−0.560	***	0.138
Hispanic	0.032		0.161	0.093		0.154	0.045		0.151
Other	−0.362	*	0.169	−0.271		0.162	−0.290		0.158
Foreign-born	−0.128		0.155	0.129		0.149	0.102		0.146
Married	−0.237	*	0.112	−0.083		0.108	−0.002		0.106
College graduate	−0.550	***	0.115	−0.454	***	0.110	−0.374	**	0.108
Employed full time	0.108		0.116	0.176		0.111	0.197		0.109
R-squared	0.206			0.270			0.299		

* refers to *p* < 0.05, ** refers to *p* < 0.01, *** refers to *p* < 0.001. B = unstandardized coefficients; SE = standard error; n = 3361.

## Data Availability

This study conducted secondary data analyses using data made available from ICPSR.
